# Efficacy and safety profile of drug-eluting beads transarterial chemoembolization by CalliSpheres® beads in Chinese hepatocellular carcinoma patients

**DOI:** 10.1186/s12885-018-4566-4

**Published:** 2018-06-08

**Authors:** Guan-Hui Zhou, Jun Han, Jun-Hui Sun, Yue-Lin Zhang, Tan-Yang Zhou, Chun-Hui Nie, Tong-Yin Zhu, Sheng-Qun Chen, Bao-Quan Wang, Zi-Niu Yu, Hong-Liang Wang, Li-Ming Chen, Wei-Lin Wang, Shu-Sen Zheng

**Affiliations:** 1 Hepatobiliary and Pancreatic Interventional Treatment Center, Division of Hepatobiliary and Pancreatic Surgery, The First Affiliated Hospital, College of Medicine, Zhejiang University, Hangzhou, 310003 Zhejiang Province China; 2 Key Laboratory of Combined Multi-organ Transplantation, Ministry of Public Health, Hangzhou, 310003 Zhejiang Province China; 3 Key Laboratory of Precision Diagnosis and Treatment for Hepatobiliary and Pancreatic Tumor of Zhejiang Province, Hangzhou, 310003 Zhejiang Province China; 4 Collaborative Innovation Center for Diagnosis Treatment of Infectious Diseases, Zhejiang University, Hangzhou, 310003 Zhejiang Province China

**Keywords:** DEB-TACE, CalliSpheres®, Hepatocellular carcinoma (HCC), Efficacy, Safety, Predictive factors

## Abstract

**Background:**

This study aimed to investigate the efficacy and safety of drug eluting beads transarterial chemoembolization (DEB-TACE) treatment by CalliSpheres® in Chinese patients with hepatocellular carcinoma (HCC) as well as the predicting factors for response.

**Methods:**

99 patients with HCC were consecutively enrolled in this study. All participants were treated by CalliSpheres® DEB-TACE. Clinical response was evaluated according to modified Response Evaluation Criteria in Solid Tumors (mRECIST) criteria. Common Terminology Criteria for Adverse Events (CTCAE) was used to assess the adverse events and liver dysfunction during and after the operation.

**Results:**

Post treatment, 16 patients (16.2%) achieved CR and 59 (59.6%) achieved PR, the ORR was 75.8%. Subgroup analysis showed that patients with higher BCLC stage were of worse CR and ORR rates, and the CR as well as ORR between patients with cTACE history and patients without cTACE history were similar. Univariate logistic regression analysis displayed that number of nodules > 3, higher BCLC stage and previous cTACE might be correlated with worse ORR but with no statistical significance. As to liver function, CTCAE grades of laboratory indexes for liver function were increased at 1 week compared to baseline and recovered to the baseline grades at 1–3 months post operation. Besides, most of the common adverse events were light and moderate in our study.

**Conclusions:**

In conclusion, DEB-TACE by CalliSpheres® was efficient and well tolerated in Chinese HCC patients, and BCLC stage, number of nodules and cTACE history were possibly correlated with treatment response.

## Background

As the second leading cause of cancer-related deaths worldwide and the predominant histological type of primary liver cancers, hepatocellular carcinoma (HCC) has attracted increasing attention for its poor prognosis due to delay in diagnosis [1]. Although in some part of the world the incidence of HCC was declined due to the identification of high-risk population, such as patients with hepatitis B, unfortunately, the global mortality is still high [2]. What is more, prevalence and mortality were especially high in East and South-East Asia, during 2012, China alone accounted for half of the HCC cases and deaths worldwide [1]. When comes to treatment, curative treatments, including resection, transplantation and local ablative therapies, could only be conducted on patients with early stage HCC, while for patients with advanced HCC the options consist of transarterial therapy, radiotherapy and chemotherapy [3].

Transarterial chemoembolization (TACE) is one major type of transarterial therapies, and the techniques of TACE include embolism of tumor-supplying vessels, which causes ischemia and necrosis of tumors, and infusion of chemotherapy agents [4]. TACE is recommended as a standard therapy for inoperable HCC patients, and conventional TACE (cTACE) is the TACE that combined embolism particles with chemotherapy drug delivery. Although cTACE is frequently applied in clinical practice, the systemic toxicity of chemotherapy drug after treatment cannot be ignored [5, 6]. The drawbacks of cTACE led to its gradual replacement by Drug-eluting beads TACE (DEB-TACE), which is a new technique for chemoembolization using microbeads with diameter ranging from 100 μm to 900 μm, providing more constant drug delivering effect and embolization of stable tumor-supplying arteries. Moreover, DEB-TACE is reported to be more effective and tolerable compared to cTACE in clinical practice [7–9]. In China, few studies have been performed to evaluate the efficacy and safety of DEB-TACE treatment for patient with HCC, and the strategy on which type of patients will benefit more from DEB-TAC is not well investigated either.

Therefore, our study aimed to investigate the efficacy and safety of DEB-TACE treatment by CalliSpheres® in Chinese patients with HCC as well as the predicting factors for response.

## Methods

### Participants

Ninety nine patients with HCC at the Hepatobiliary and Pancreatic Interventional Treatment Center of our hospital from 18^th^November, 2015 to 19th October, 2016 were consecutively enrolled in this study. Patients were included if they met the criteria as follows: (1) diagnosed as HCC according to the criteria of the American Association for the Study of the Liver Diseases (AASLD) [10]; (2) age above 18 years; (3) Child Pugh stage A or B; (4) Eastern Cooperative Oncology Group (ECOG) score no more than 2; (5) about to receive DEB-TACE treatment on demand; (6) The result of laboratory examinations should meet the following criteria: platelet count > 50 × 10^9^/L, haemoglobin > 8.0 g/dl, prolongation of the prothrombin time < 6 s, albumin > 2.8 g/dl, bilirubin < 51 μmol/L, alanine and aspartate aminotransferase < 3 times the upper limit of the normal range, serum creatinine < 1.5 times the upper limit of the normal range. And the exclusion criteria were as follows: (1) severe liver or renal failure; (2) known allergic to or with contraindications of the chemoembolization reagent in this study; (3) intrahepatic arterial-portal fistula or intrahepatic arteriovenous fistula; (4) uncontrolled ascites; (5) hepatic encephalopathy; (6) other primary malignancies; (7) women in the duration of pregnancy or lactation.

This study was approved by the Medical Ethics Committee of our hospital and followed the Declaration of Helsinki principles. All participants signed the informed consents.

### Chemoembolization procedure

Doxorubicin and Epirubicin were used as chemoembolization reagents, CalliSpheres® (Jiangsu Hengrui Medicine Co., Ltd., Jiangsu, China) beads (100-300 μm) were loaded with the above reagents, and the loading dose of reagents ranged from 50 mg to 100 mg. The loading process was performed as follows: (1) chemoembolization reagents were dissolved at the concentration of 20 mg/ml; (2) one vial of CalliSpheres® beads was shaken up and the supernatant was extracted, after that the beads and the chemoembolization solution were mixed by a tee joint; (3) then the mixed solution was shaken up and stand for 30 min at room temperature, subsequently, the non-ionic contrast agent was added and the mixed solution was stand for another 5 min for further application.

Before embolization, angiography guided by computerized tomography (CT) was performed to check whether there was arteriovenous shunt or not as well as to detect the arterial feeders of tumors. Additionally, all embolizations were conducted under topical anesthesia. Subsequently, the tumor feeding arteries were catheterized by 2.4 French microcatheters (Merit Maestro, Merit Medical System, Inc., Utah, USA) under angiogram. After microcatheters were placed, the mixed solution of CalliSpheres® beads and chemoembolization reagents was injected at the speed of 1 ml/min. And the injection was stopped on the condition of stasis flow of contrast agent existed. Afterward, a second angiography was conducted after 5 min, and embolization was continued if blushed tumors still appeared. Until all blushed tumors disappeared, the microcatheters were pulled out and the embolization was completed. If one vial of CalliSpheres® beads was used and the embolization was not completed, another vial would be utilized to reach the embolization endpoint. For those patients with bilobar disease, the DEB-TACE was performed on both lobes of the patient.

### Assessments

Comprehensive baseline characteristics of patients were documented and listed, including (1) demographic characteristics: age, and gender; (2) history: hepatitis history, cirrhosis history and drink; (3) clinicopathological characteristics: tumor distributions, number of nodules, largest nodule size, portal vein invasion, hepatic vein invasion, Eastern Cooperative Oncology Group (ECOG) performance status, Barcelona Clinic Liver Cancer (BCLC) stage; (4) laboratory indexes: white blood cell (WBC), red blood cell (RBC), absolute neutrophil count (ANC), hemoglobin (HB), platelet (PLT), albumin (ALB), total protein (TP), total bilirubin (TBIL), total bile acid (TBA) alanine aminotransferase (ALT), aspartate aminotransferase (AST), alkaline phosphatase (ALP), blood creatinine (BCr), blood urea nitrogen (BUN), alpha fetoprotein (AFP), carcino-embryonic antigen (CEA), carbohydrate antigen199 (CA199); (5) treatment history: cTACE, surgery, systematic chemotherapy, radiofrequency ablation and targeted therapy; (6) chemoembolization reagents: Epirubicin.

In addition, the levels for dividing normal and abnormal laboratory indexes was 4~ 10 × 10^9^/L for WBC, 4.09~ 5.74 × 10^12^/L (male) or 3.68~ 5.13 × 10^12^/L (female) for RBC, 50%~ 70% for ANC, 131~ 172 g/L (male) or 113~ 151 g/L (female) for Hb, 83~ 303 × 10^9^/L (male) or 101~ 320 × 10^9^/L (female) for PLT, 35~ 55 g/L for ALB, 64~ 83 g/L for TP, 0~ 21 μmol/L for TBIL, 1~ 12 μmol/L for TBA, 5~ 40 U/L for ALT, 8~ 40 U/L for AST, 40~ 150 U/L for ALP, 59~ 104 μmol/L (male) or 45~ 84 μmol/L (female) for BCr, 2.9~ 8.2 mmol/L for BUN, 0~ 20.0 ng/ml for AFP, 0~ 5.0 ng/ml for CEA and 0~ 37.0 U/mL for CA199.

Clinical response of patients post treatment was assessed by CT or magnetic resonance image (MRI) at 1–3 months post treatment, according to the modified Response Evaluation Criteria in Solid Tumors (mRECIST) criteria [11]: (1) complete response (CR): disappearance of any intratumoral arterial enhancement in all target nodules; (2) partial response (PR): at least a 30% decrease in the sum of diameters of viable (enhancement in the arterial phase) target nodules, taking as reference the baseline sum of the diameters of target nodules; (3) stable disease (SD): any cases that do not qualify for either PR or progressive disease; (4) progressive disease (PD): an increase of at least 20% in the sum of the diameters of viable (enhancing) target nodules, taking as reference the smallest sum of the diameters of viable (enhancing) target nodules recorded since treatment started. Overall response rate (ORR) was defined as the percentage of patients or treated nodules reached CR and PR. In addition, each treatment response assessment was performed through central independent review in our hospital. And for those patients received multiple cycles of DEB-TACE procedures, we only assessed treatment response of the first cycle DEB-TACE procedure.

Adverse events during and post treatment were recorded, and laboratory indexes of patients pre and post treatment were documented. The severity of pain was graded by pain visual analogue scale (VAS) score [12], and the vomiting grade was determined by the vomiting times. Additionally, liver damage of patients was evaluated according to laboratory indexes related to liver function, and the severity of liver damage was assessed by the Common Terminology Criteria for Adverse Events (CTCAE) version 4.0 made by National Cancer Institute (NCI) [13].

### Statistics

Statistical analysis was performed using SPSS 22.0 software (IBM, USA) and Office 2010 software (Microsoft, USA). Data was presented as count, count (%), mean ± standard deviation or median (25th–75th). Comparison between groups was determined by t test or Chi-square test. Wilcoxon signed rank sum test was used for comparing the difference of liver damage in patients pre and post treatment. Univariate and multivariate logistic regression were performed to evaluate the predicting factors for ORR. *P* < 0.05 was considered significant.

## Results

### Study flow

**Fig. 1:**
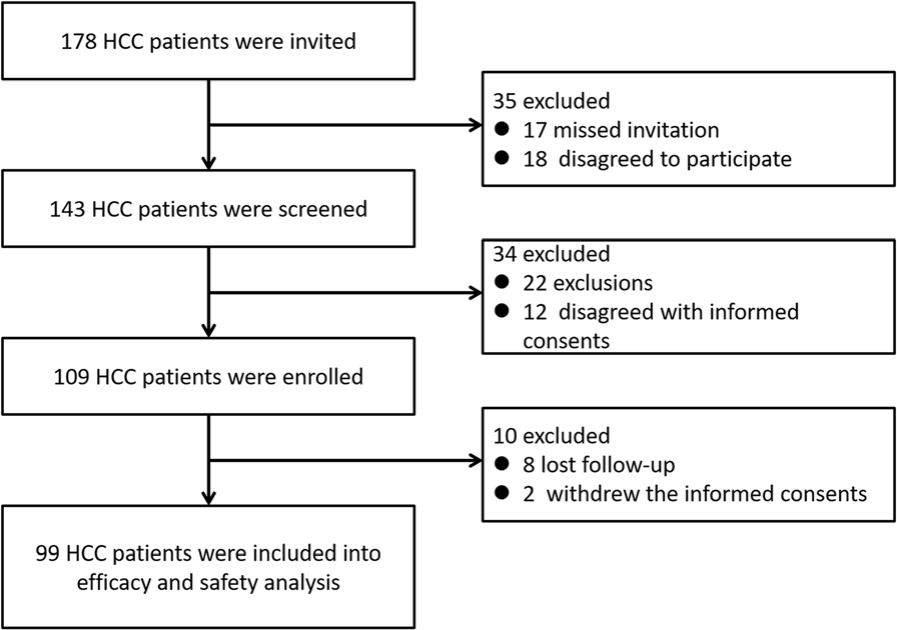
Study flow

As displayed in Fig. 1, 178 HCC patients were initially invited and 35 patients were excluded due to missing the invitation (17) and disagreed to participate (18). Subsequently, the remaining 143 HCC patients were screened and after which 34 patients were excluded (22 exclusions and 12 disagreed with informed consents), which led to 109 HCC patients were enrolled in our study. Afterwards, 10 patients were excluded, among them 8 patients were lost follow-up and 2 patients withdrew the informed consents.

### Baseline characteristics of HCC patients

Baseline characteristics of patients were listed in Table 1, mean age of patients was 57.98 ± 10.33 years, and there were 89 males and 10 females. 94 (94.9%) patients were with hepatitis B and 2 (2.1%) were with hepatitis C. Additionally, 55 (55.6%) patients were combined with cirrhosis. The median tumor distribution was 20.0 (5.0–30.0) %, and the largest nodule size was 4.3 (2.1–8.3) cm. There were 29 (29.3%) patients had portal vein invasion in our study, among whom the number of patients with main portal vein invasion, first branch invasion and second branch invasion were 8 (8.1%), 8 (8.1%) and 13 (13.1%). In addition, the number of patients with ECOG 0 and 1 were 70 (70.7%) and 29 (29.3%), respectively. Liver function of patients at baseline was assessed by Child-pugh classification, and the number of patients with Child-pugh stage A and B were 91 (91.2%) and 8 (8.1%). In addition, 24 (24.2%) patients were BCLC stage A, 35 (35.4%) patients were BCLC stage B and 40 (40.4%) patients were BCLC C stage, and among all the patients were BCLC C stage 9 (22.5%) of them had metastasis. Besides, 66 (66.7%) patients were with surgery history, and the number of patients with cTACE history was 39 (39.4%) with median cTACE times of 1 (1–2). Other history, clinicopathological characteristics, laboratory indexes and chemoembolization reagents information were presented in Table 1.

**Table 1:** Baseline characteristics

Parameters	Patients (*N* = 99)
Age (years)	57.98 ± 10.33
Gender (Male/Female)	89/10
Hepatitis history	
No hepatitis history (n/%)	3 (3.1)
HBV (n/%)	94 (94.9)
HCV (n/%)	2 (2.1)
Drink (n/%)	43 (43.4)
Cirrhosis (n/%)	55 (55.6)
Tumor distribution^a^ (%)	20.0 (5.0–30.0)
Number of nodules	
1 (n/%)	30 (30.1)
> 1 (n/%)	69 (69.9)
< =3 (n/%)	54 (54.5)
> 3 (n/%)	45 (45.5)
Largest nodule size (cm)	4.3 (2.1–8.3)
Portal vein invasion (n/%)	29 (29.3)
Main portal vein invasion (n/%)	8 (8.1)
First branch invasion (n/%)	8 (8.1)
Second branch invasion (n/%)	13 (13.1)
Hepatic vein invasion (n/%)	17 (17.2)
ECOG performance status	
0 (n/%)	70 (70.7)
1 (n/%)	29 (29.3)
Child-pugh Stage	
A (n/%)	91 (91.2)
B (n/%)	8 (8.1)
BCLC Stage	
A (n/%)	24 (24.2)
B (n/%)	35 (35.4)
C (n/%)	40 (40.4)
Laboratory Indexes	
WBC abnormal (n/%)	31 (31.3)
RBC abnormal (n/%)	16 (16.2)
ANC abnormal (n/%)	26 (26.3)
HB abnormal (n/%)	28 (28.3)
80~ 100 g/L	5 (5.1)
> 100 g/L	23 (23.2)
PLT abnormal (n/%)	28 (28.3)
50~ 70 × 10^9^/L	19 (19.2)
> 70 × 10^9^/L	9 (9.1)
ALB abnormal (n/%)	10 (10.1)
TP abnormal (n/%)	20 (20.2)
TBIL abnormal (n/%)	17 (17.2)
21~ 34 μmol/L	13 (13.1)
34~ 51 μmol/L	4 (4.0)
TBA abnormal (n/%)	29 (29.3)
ALT abnormal (n/%)	22 (22.2)
AST abnormal (n/%)	31 (31.3)
1~ 2ULN	25 (25.2)
2~3ULN	6 (6.1)
ALP abnormal (n/%)	19 (19.2)
BCr abnormal (n/%)	10 (10.1)
BUN abnormal (n/%)	9 (9.1)
AFP abnormal (n/%)	55 (55.6)
20.0~ 400 ng/ml	21 (21.2)
> 400 ng/ml	34 (34.3)
CEA abnormal (n/%)	12 (12.1)
CA199 abnormal (n/%)	19 (19.2)
Previous treatment	
cTACE (n/%)	39 (39.4)
cTACE times	1 (1~ 2)
Surgery (n/%)	66 (66.7)
Systematic chemotherapy (n/%)	5 (5.1)
Radiofrequency ablation (n/%)	17 (17.2)
Targeted therapy (n/%)	1 (1.0)
Chemoembolization reagents	
Adriamycin (n/%)	4 (4.1)
Epirubicin (n/%)	95 (95.9)

Data was presented as count (%), mean ± standard deviation or median (25th–75th)

^a^Tumor distribution: percentage of tumor in the whole liver. *HBV* Hepatic b virus, *HCV* Hepatic c virus, *ECOG* Eastern Cooperative Oncology Group, *BCLC* Barcelona Clinic Liver Cancer, *WBC* While blood cell, *RBC* Red blood cell, *ANC* Absolute neutrophil count, *HB* Hemoglobin, *PLT* Platelet, *ALB* Albumin, *TP* Total protein, *TBIL* Total bilirubin, *TBA* Total bile acid, *ALT* Alanine aminotransferase, *AST* Aspartate aminotransferase, *ALP* Alkaline phosphatase, *BCr* Blood creatinine, *BUN* Blood urea nitrogen, *AFP* Alpha fetoprotein, *CEA* Carcino-embryonic antigen, *CA199* Carbohydrate antigen199, *cTACE* Conventional transarterial chemo-embolization

### Treatment response of patients post DEB-TACE treatment

Post treatment, the ORR was 75.8%, among which 16 (16.2%) patients achieved CR and 59 (59.6%) achieved PR (Table 2). In terms of clinical response of treated nodules, the ORR of treated nodules were 74.3%, in which 46 (25.1%) nodules achieved CR and 90 (49.2%) nodules achieved PR. Among the nodules achieved PR, 45 (50.0%) nodules reached necrosis rate more than 80%, 32 (35.6%) nodules reached necrosis rate ranging from 50 to 80% and 13 (14.4%) nodules reached necrosis rate less than 50% (Table 3). In addition, the mean necrosis rate was (66.68 ± 19.37) %.

**Table 2:** Clinical response of patients and nodules post treatment

Parameters	Patients (*N* = 99)	Nodules (*N* = 183)
CR (n/%)	16 (16.2)	46 (25.1)
PR (n/%)	59 (59.6)	90 (49.2)
ORR (n/%)	75 (75.8)	136 (74.3)
SD (n/%)	10 (10.1)	17 (9.3)
PD (n/%)	14 (14.1)	30 (16.4)

Data was presented as count (%)

*CR* Complete response, *PR* Partial response, *ORR* Overall response rate, *SD* Stable disease, *PD* Progress disease

**Table 3:** Necrosis rate of nodules reached PR

Parameters	Nodules (*N* = 90)
Total necrosis rate (%)	66.68 ± 19.37
Necrosis rate 80%~ (n/%)	45 (50.0)
Necrosis rate 50%~ 80% (n/%)	32 (35.6)
Necrosis rate ~ 50% (n/%)	13 (14.4)

Data was presented as mean ± standard deviation or count (%)

*PR* Partial response

### The difference of treatment response in patients categorized by BCLC stage and cTACE history

**Fig. 2:**
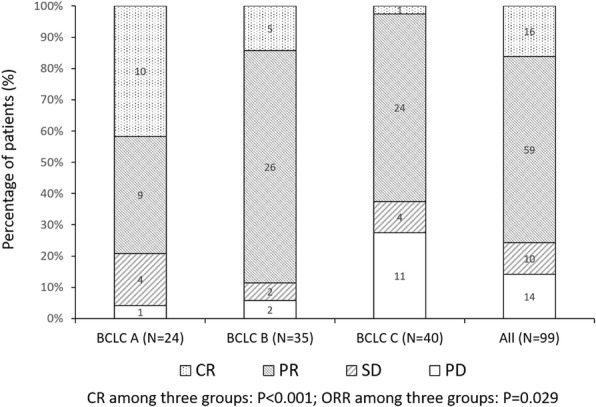
The clinical response of patients in different BCLC stage. Comparison among groups was determined by Chi-square test. *P* < 0.05 was considered significant

**Fig. 3:**
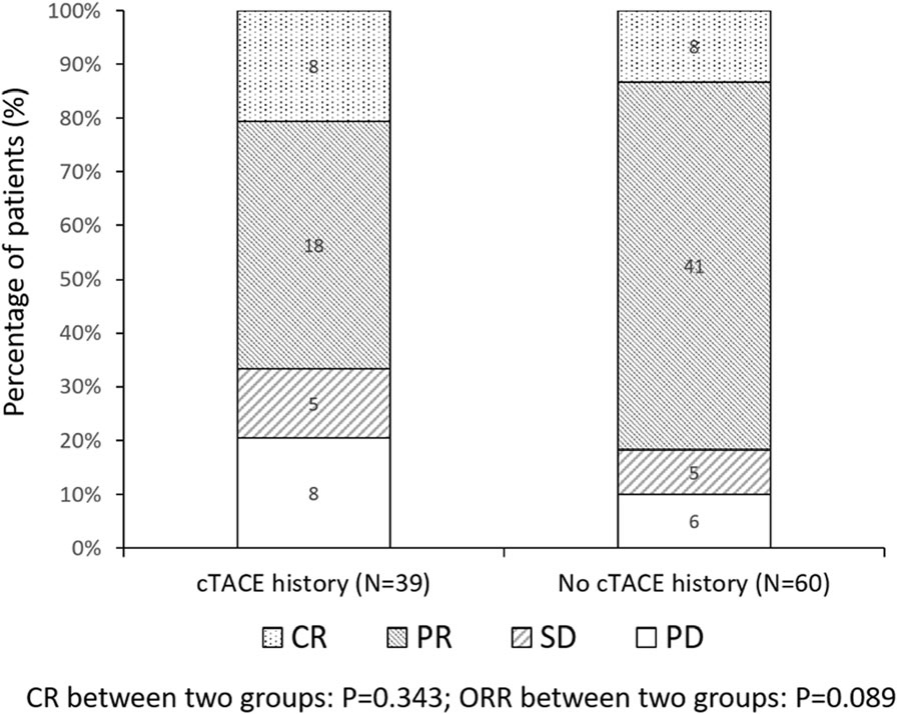
The clinical response of patients with or without previous cTACE treatment. Comparison among groups was determined by Chi-square test. *P* < 0.05 was considered significant

The differences of treatment response in patients with various BCLC stages and different cTACE histories were presented in Figs. 2 and 3, which exhibited that patients with higher BCLC stage were of worse CR and ORR rate (*P* < 0.001 and *P* = 0.029, respectively) (Fig. 2), and no difference of CR and ORR was found between patients with or without cTACE treatment history (*P* = 0.343 and *P* = 0.089, respectively) (Fig. 3).

### Subgroup analysis of ORR

Besides BCLC stage and cTACE history, we also analyzed the differences of ORR in patients divided by other comprehensive baseline characteristics, which were listed in Table 4. And the results showed that the number of nodules was associated with ORR in patients post treatment. To be exact, the ORR of patients with multiple nodules tended to be lower than that of patients with single nodule (*P* = 0.095), and patients with > 3 nodules were likely to achieve worse ORR compared to patients with number of nodules <= 3 (*P* = 0.054). However, the differences were both not statistically significant.

**Table 4:** Subgroups analysis of ORR achievement

Parameters	ORR (*n* = 75)	Not ORR (*n* = 24)	*P* value
Age (years)	58.27 ± 10.09	57.04 ± 11.05	
Gender Male (n/%)	68 (76.4)	21 (23.6)	0.654
HBV (n/%)	71 (75.5)	23 (24.5)	0.820
HCV (n/%)	1 (50.0)	1 (50.0)	0.390
Drink (n/%)	33 (76.7)	10 (23.3)	0.841
Cirrhosis (n/%)	41 (74.5)	14 (25.5)	0.753
Tumor distribution^a^			0.765
> =20% (n/%)	38 (74.5)	13 (25.5)	
< 20 (n/%)	37 (77.1)	11 (22.9)	
Number of nodules			
1 (n/%)	26 (86.7)	4 (13.3)	0.095
> 1 (n/%)	49 (71.0)	20 (29.0)	
< =3 (n/%)	45 (83.3)	9 (16.7)	0.054
> 3 (n/%)	30 (66.7)	15 (33.3)	
Largest nodule size > 3 cm (n/%)	54 (78.3)	15 (21.7)	0.278
Largest nodule size > 5 cm (n/%)	33 (75.0)	11 (25.0)	0.875
Portal vein invasion (n/%)	19 (65.5)	10 (34.5)	0.126
Main portal vein invasion (n/%)	3 (37.5)	5 (62.5)	0.173
First branch invasion (n/%)	4 (50.0)	4 (50.0)	
Second branch invasion (n/%)	10 (76.9)	3 (23.1)	
Hepatic vein invasion (n/%)	12 (70.6)	5 (29.4)	0.585
ECOG performance status			0.595
0 (n/%)	52 (74.3)	18 (25.7)	
1 (n/%)	23 (79.3)	6 (20.7)	
Child-pugh stage			0.419
A (n/%)	68 (74.7)	23 (25.3)	
B (n/%)	7 (87.5)	1 (12.5)	
BCLC stage			0.029
A (n/%)	19 (79.2)	5 (20.8)	
B (n/%)	31 (88.6)	4 (11.4)	
C (n/%)	25 (62.5)	15 (37.5)	
AFP			0.432
Abnormal (n/%)	40 (72.7)	15 (27.3)	
Normal (n/%)	35 (79.5)	9 (20.5)	
Previous cTACE			0.089
Yes (n/%)	26 (66.7)	13 (33.3)	
No (n/%)	49 (81.7)	11 (18.3)	
Previous surgery			0.136
Yes (n/%)	53 (80.3)	13 (19.7)	
No (n/%)	22 (66.7)	11 (33.3)	
Previous systematic chemotherapy			0.820
Yes (n/%)	4 (80.0)	1 (20.0)	
No (n/%)	71 (75.5)	23 (24.5)	
Previous radiofrequency ablation			0.585
Yes (n/%)	12 (70.6)	5 (29.4)	
No (n/%)	63 (76.8)	19 (23.2)	
Previous targeted therapy (n/%)			0.562
Yes (n/%)	1 (100.0)	0 (0.0)	
No (n/%)	74 (75.5)	24 (24.5)	
Chemoembolization reagent			0.248
Adriamycin (n/%)	4 (100.0)	0 (0.0)	
Epirubicin (n/%)	71 (74.7)	24 (25.3)	

Data was presented as count (%) or mean ± standard deviation

Comparison between groups was determined by t test or Chi-square test. *P* < 0.05 was considered significant. ^a^Tumor distribution: percentage of tumor in the whole liver

*HBV* Hepatic b virus, *HCV* Hepatic c virus, *ECOG* Eastern Cooperative Oncology Group, *BCLC* Barcelona Clinic Liver Cancer, *AFP* Alpha fetal protein, *cTACE* Conventional transarterial chemo-embolization

### Predictive factors analysis of ORR

To explore the predictive factors for ORR in patients, logistic regression analysis was performed. As shown in Table 5, univariate logistic regression analysis displayed that number of nodules > 3 (*P* = 0.058), higher BCLC stage (*P* = 0.073) and previous cTACE (*P* = 0.093) were likely to be correlated with worse ORR achievement post treatment. And the multivariate logistic regression analysis was performed to evaluate the factors in the univariate logistic regression analysis with *P* < 0.1, which exhibited that none of those three factors could independently predict the ORR (all *P* > 0.05).

**Table 5:** Factors affecting ORR achievement by logistic regression analysis

Parameters	Univariate logistic regression				Multivariate logistic regression			
	*P* value	OR	95% CI		*P* value	OR	95% CI	
			Lower	Higher			Lower	Higher
Age > =60 years	0.328	0.630	0.250	1.590	–	–	–	–
Gender (male)	0.655	1.388	0.329	5.847	–	–	–	–
HBV	0.821	0.772	0.082	7.258	–	–	–	–
HCV	0.415	0.311	0.019	5.168	–	–	–	–
Drink	0.841	1.100	0.434	2.790	–	–	–	–
Cirrhosis	0.753	0.861	0.340	2.183	–	–	–	–
Tumor distribution > = 20%	0.765	0.993	0.384	2.566	–	–	–	–
Number of nodules > 1	0.103	0.377	0.117	1.219	–	–	–	–
Number of nodules > 3	0.058	0.400	0.155	1.031	0.381	0.618	0.211	1.813
Largest nodule size > 3 cm	0.270	1.692	0.664	4.313	–	–	–	–
Largest nodule size > 5 cm	0.785	0.879	0.349	2.216	–	–	–	–
Portal vein invasion	0.130	0.475	0.181	1.246	–	–	–	–
Hepatic vein invasion	0.526	0.686	0.213	2.204	–	–	–	–
ECOG = 1 (vs. 0)	0.596	1.327	0.466	3.778	–	–	–	–
Child-pugh stage B (vs. stage A)	0.432	2.368	0.276	20.285	–	–	–	–
Higher BCLC stage	0.073	0.560	0.297	1.056	0.178	0.600	0.285	1.262
AFP abnormal	0.432	0.745	0.290	1.918	–	–	–	–
Previous cTACE	0.093	0.449	0.177	1.142	0.095	0.433	0.162	1.158
Previous surgery	0.139	2.038	0.793	5.241	–	–	–	–
Previous systematic chemotherapy	0.821	1.296	0.138	12.186	–	–	–	–
Previous radiofrequency ablation	0.586	0.724	0.226	2.315	–	–	–	–
Previous targeted therapy	–	–	–	–	–	–	–	–
Epirubicin (vs. Adriamycin)	–	–	–	–	–	–	–	–

Data was presented as P value, OR (odds ratio) and 95% CI. Factors affecting ORR achievement were determined by univariate logistic regression analysis, while all factors with *P* value no less than 0.1 were further detected by multivariate logistic regression analysis. P Value < 0.05 was considered significant. Previous targeted therapy and Epirubicin (vs Adriamycin) were not able to be analyzed due to lack of effective events. BCLC score was defined as 0-Stage A, 1-Stage B, 2-Stage C to be analyzed in logistic model. *HBV* Hepatic b virus, *HCV* Hepatic c virus, *ECOG* Eastern Cooperative Oncology Group, *BCLC* Barcelona Clinic Liver Cancer, *AFP* Alpha fetal protein, *cTACE* Conventional transarterial chemo-embolization

### Liver function change before and after DEB-TACE

Laboratory indexes of liver function pre and post treatment were evaluated, which showed that the CTCAE grades of ALB, TBIL, ALT and AST levels of patients at baseline were only grade 0, 1 and 2, among which the grade 0 was the most prominent CTCAE grade (Table 6). Post treatment, the CTCAE grades of ALB, TBIL, ALT and AST levels were increased at 1 week compared to those at baseline (all *P* < 0.001), while were recovered at 1–3 months (*P* = 0.134, *P* = 0.643, *P* = 0.614 and *P* = 0.218, respectively).

**Table 6:** Liver function impairment grade of patient pre and post DEB-TACE treatment

Parameters	Baseline (*N* = 99)	1 week post treatment (*N* = 91)	1–3 month post treatment (*N* = 88)	*P* value^*^	*P* value^#^
ALB (n)				< 0.001	0.134
Grade 0	8	59	76		
Grade 1	10	26	11		
Grade 2	2	6	1		
Grade 3	0	0	0		
Grade 4	0	0	0		
TBIL (n)				< 0.001	0.643
Grade 0	67	26	59		
Grade 1	23	36	21		
Grade 2	9	25	6		
Grade 3	0	4	2		
Grade 4	0	0	0		
ALT (n)				< 0.001	0.614
Grade 0	76	32	63		
Grade 1	20	37	23		
Grade 2	3	8	2		
Grade 3	0	13	0		
Grade 4	0	1	0		
AST (n)				< 0.001	0.218
Grade 0	62	25	54		
Grade 1	35	42	29		
Grade 2	2	12	3		
Grade 3	0	10	2		
Grade 4	0	1	0		

Data was presented as count. Comparison among groups was determined by Wilcoxon signed rank sum test. *P* < 0.05 was considered significant. ^*^P value of liver function related biochemical indexes of patients from baseline to 1 week post treatment. ^#^*P* value of liver function related biochemical indexes of patients from baseline to 1–3 month post treatment. *ALB* Albumin, *TBIL* Total bilirubin, *ALT* Alanine aminotransferase, *AST* Aspartate aminotransferase

### Safety profile of common adverse events during and post treatment

As listed in Table 7, pain, vomiting and hypertension were the most common adverse events during and post treatment (<=24 h). Precisely, the number of patients presented with light, moderate and severe pain were 63 (63.6%), 30 (30.3%) and 2 (2.01%), indicating that most patients were with light to moderate pain during and post treatment (<=24 h), and 4 (4.0%) patients showed no sign of pain. For vomiting, 83 (83.8%) patients did not vomit, and the number of patients presented with Grade 1 and Grade 2 vomiting were 13 (13.1%) and 3 (3.0%), respectively. Only 16 (16.2%) patients presented with hypertension. In addition, fever was the most common adverse event post treatment (24-72 h). Most patients had no fever (23 (23.2%)), low-grade (36 (36.4%)) and median-grade (31 (31.3%)) fever, only 9 (9.1%) patient was with high-grade fever.

**Table 7:** Adverse events of DEB-TACE treatment

Parameters	HCC Patients (*N* = 99)
During and post operation (<=24 h)	
Pain^a^	
No pain^a^ (n/%)	4 (4.0)
Light pain^a^ (n/%)	63 (63.6)
Moderate pain^a^ (n/%)	30 (30.3)
Severe pain^a^ (n/%)	2 (2.01)
Vomiting^b^	
No vomiting^b^ (n/%)	83 (83.8)
Grade 1^b^ (n/%)	13 (13.1)
Grade 2^b^ (n/%)	3 (3.0)
Hypertension (n/%)	16 (16.2)
Post operation (24 h–72 h)	
Fever	
No fever (n/%)	23 (23.2)
Low-grade fever (n/%)	36 (36.4)
Median-grade fever (n/%)	31 (31.3)
High-grade fever (n/%)	9 (9.1)

Data was presented as count (%)

*Pain VAS* Pain visual analogue scale

^a^The severity of pain was calculated by pain VAS: No pain: pain VAS score = 0; Light pain: pain VAS score = 1–3; Moderate pain: pain VAS score = 4–6; Severe pain: pain VAS score = 7–10. ^b^Grade 1: times of vomiting =1–2; Grade 2: times of vomiting = 3–5

### Change of child Pugh score and the impact of cTACE history and portal vein invasion

As presented in Table 8, post treatment, 3 (3.0%) patients had a decline of Child Pugh score ≥ 2 points at 1 week and 60 (60.6%) patients had the Child Pugh score returned to baseline at 3 months.

**Table 8:** Change of Child-pugh score post DEB-TACE

Parameter	Child-pugh score declined ≥2 points at 1 week	Child-pugh score returned to baseline at 3 months
Count (%)	3 (3.0)	60 (60.6)

Data was presented as count (percentage)

Subgroup analysis revealed that previous cTACE history (*P* = 0.827) and portal vein invasion (*P* = 0.553) had no impact on the percentage of patients had Child Pugh score declining ≥2 points at one-week post DEB-TACE (Table 9). In addition, previous cTACE history (*P* = 0.634) and portal vein invasion (*P* = 0.425) have no influence on the proportion of patients had Child Pugh score return to baseline at 3 months after DEB-TACE as well.

**Table 9:** Subgroup analysis for Change of Child-pugh score post DEB-TACE

Parameter	Child-pugh score declined ≥2 points at 1 week			Child-pugh score returned to baseline at 3 months		
	Yes	No	*P* value	Yes	No	*P* value
Previous cTACE (n/%)	1 (2.6)	38 (97.4)	0.827	25 (64.1)	14 (35.9)	0.634
Portal vein invasion (n/%)	0 (0.0)	29 (100.0)	0.553	16 (55.2)	13 (44.8)	0.425

Data was presented as count (%). Comparison was determined by Chi-square test. cTACE: conventional transarterial chemo-embolization

Univariate logistic regression analysis revealed that previous cTACE history (*P* = 0.828) and portal vein invasion (*P* = 0.998) were not correlated with Child-pugh declined by 2 points after 1 week of DEB-TACE (Table 10), and multivariate logistic regression illuminated that cTACE history (*P* = 0.735) and portal vein invasion (P = 0.998) were not independent predictive factors. And univariate as well as multivariate logistic regression analyses elucidated that cTACE history (*P* = 0.635 and *P* = 0.456, respectively) and portal vein invasion (*P* = 0.426 and *P* = 0.699, respectively) were not correlated or independently associated with Child Pugh score change at 3 months post DEB-TACE (Table 11).

**Table 10:** Logistic regression analysis for Child-pugh score declined ≥2 score at 1 week of post operation

Parameters	Univariate logistic regression				Multivariate logistic regression			
	*P* value	OR	95% CI		*P* value	OR	95% CI	
			Lower	Higher			Lower	Higher
Previous cTACE	0.828	0.763	0.067	8.713	0.735	0.655	0.057	7.582
Portal vein invasion	0.998	0.000	0.000	–	0.998	0.000	0.000	–

Data was presented as P value, OR (odds ratio) and 95% CI. P Value < 0.05 was considered significant. *cTACE* conventional transarterial chemo-embolization

**Table 11:** Logistic regression analysis of Child-pugh score returned to baseline after 3 months of operation

Parameters	Univariate logistic regression				Multivariate logistic regression			
	*P* value	OR	95% CI		*P* value	OR	95% CI	
			Lower	Higher			Lower	Higher
Previous cTACE	0.635	1.224	0.531	2.823	0.456	0.713	0.294	1.733
Portal vein invasion	0.426	0.699	0.290	1.688	0.699	1.181	0.508	2.744

Data was presented as P value, OR (odds ratio) and 95% CI. P Value < 0.05 was considered significant. *cTACE* conventional transarterial chemo-embolization

## Discussion

In our study, we discovered that: (1) DEB-TACE treatment for HCC patients was of good efficacy: the CR rate was 16.2%, PR rate was 59.6% and the ORR was 75.8%; (2) subgroup analysis illustrated that patients with higher BCLC stage were of lower CR and ORR, and logistic regression analysis elucidated that more than 3 nodules, higher BCLC stage and previous cTACE history might be associated with worse ORR; (3) liver function of patients recovered at 1–3 months after a rapid worsening at 1 week post treatment. And DEB-TACE was well tolerated in HCC patients, only light and moderate common adverse events in our study were observed.

Drug eluting bead (DEB) plays a crucial role in the technique of DEB-TACE, the DEBs are capable of being impregnated with anti-tumor drugs and continuously delivering the drugs, accomplishing more stable and constant drug concentration [4]. In this study, the CalliSpheres® beads were used in the DEB-TACE procedure, which is the first microbead product in China. The CalliSpheres® beads is a class of mircrobeads made of polyvinyl alcohol hydrogel with different ranges of diameters, and an in vivo experiment elucidates that the doxorubicin concentration in rabbit livers using CalliSpheres® beads was higher than using arterial infusion with a relatively greater delivery distance of 200 μm from the bead edge for over a month [14]. According to previous studies, DEB-TACE shows good efficacy in treating HCC patients, the ORR ranges from 35%~ 84% [15–18]. A study that was conducted on patients with HCC receiving DEB-TACE therapy elucidates a CR rate of 58% and PR rate of 31%, which is of better CR rate compared to ours [15]. While in the study of Rahman FA et al., 17% patients achieve CR and 22% patients achieves PR, the ORR is 39%, which is lower than ours due to the lower PR rate [16]. And a study that was performed in Korea shows that the CR rate is 32.1%, PR rate is 28.3% and the ORR is 60.4% [18]. The difference in response rate might result from that: (1) the different stages of HCC patients enrolled; (2) DEB-TACE is used as first-line treatment in some of their studies, which might result in a higher response rate; (3) the variant sample sizes among studies may cause different response rate.

Although DEB-TACE is recommended as a standard therapy for HCC patients in BCLC B stage, due to this was an observational study in which the patients enrolled were not selected, thus there were 24% of our patients who were in BCLC stage A. Those patients did not receive DEB-TACE due to they were not qualified, for example, the patients with multiple lesions, poor liver function or financial issues. Besides, there are several studies indicate DEB-TACE could be used for HCC patients in early stage. A single-center, single-arm, retrospective study with a cohort of 421 HCC patients received DEB-TACE also includes 20.9% patients in BCLC stage A and discloses good overall response rates at 3 months (94.5%) and 6 months (99.5%) [19]. Another retrospective cohort study also enrolls 39.4% patients in BCLC stage A, who are not treated by standard treatments or alternative therapies due to ineligible for ablation and post treatment recurrence, shows that the median survival is 54.2 months for patients in BCLC stage A [20].

Personalized treatment of DEB-TACE has now become more and more concerned among oncologists. Although criteria have been made for the prognosis of HCC patients, such as BCLC stage for risk stratify and Child-pugh class for liver function staging, predictive factors for treatment response of DEB-TACE are still not well established. C-arm CT and volume perfusion CT (VPCT) for blood volume assessment have been reported by a prior study that they are able to predict the midterm tumor response of patients [21]. Furthermore, a study illustrates that tumor heterogeneity and tumor enhancement more than 50% are correlated with CR in HCC patients after DEB-TACE therapy [22]. And in the study of Kokabi N et al., they discover that the apparent diffusion coefficients (ADC) at baseline < 0.83 × 10^− 3^ mm^2^/s is a predictive factor for both clinical response and survival of HCC patients after DEB-TACE [23]. In our study, patients with higher BCLC stage were of worse CR and ORR, patients in BCLC stage A and B both presented with impressive treatment response rates, while patients in BCLC stage C were of extremely poor response rate. The results in regard to BCLC stage in this study might be as a consequence of that BCLC staging system assesses the liver function, tumor distribution and patients’ physical status, playing a prognostic role in the management of HCC patients [24]. In the study of Kao WY et al., 1265 treatment-naive HCC patients are enrolled and the result shows that patients in stage 0 and A1 have markedly higher overall survival rates than those in stages A2-A4, however, they do not compare the efficacy among patients in different BCLC stages [25]. For the predictive value of BCLC stage for treatment response, a previous study illustrates that HCC patients with BCLC stage B and C have similar treatment response after chemoembolization therapy [26].

Besides BCLC stage, our study also observed that patients with multiple nodules and previous cTACE history had the trend of exhibiting worse ORR. Multifocal had been well established as a factor for predicting worse prognosis in patients with HCC [27–29]. And the number of nodules > 3 has been reported as a predicting factor for poor survival of HCC patients after resection [29]. Those results discovered by former studies indicated that the number of nodules may be of good prognostic value, which is partly in line with ours. As for patients with cTACE history, those patients had worse ORR in our study might result from that patients were less sensitive to the DEB-TACE treatment after previous cTACE treatment.

Moreover, the liver function of patients deteriorated rapidly and recovered at 1–3 months after the procedure in our study. A former study has evaluated the liver damage after DEB-TACE, in their study, the liver damage is assessed by image and in 114 patients with HCC, the results validates that the number of patients occurs with global liver damage, overall biliary injuries, intrahepatic biloma and portal vein thrombosis are 42 (36.8%), 37 (32.5%), 19 (16.7%) and 5 (4.4%) [30]. Hepatic artery injury is also a common adverse event in DEB-TACE treatment, it is reported that in 54 HCC patients received DEB-TACE treatment, the number of HCC patients occurred with grade I, II and III hepatic artery injuries are 13 (24.1%), 10 (18.5%) and 31 (57.4%) [31]. Compared to those two former studies, the liver damage in our study was relatively lighter, and the difference might result from that the global liver function of patients at baseline were variant among studies. And the reason why patients had expeditious liver function damage at 1 week might be due to the liver damage caused by the operative injury. Moreover, the recovery of liver function at 1–3 months may be because that patients in our study were mostly with intermediate liver function before operation, which indicated most patients still maintained their self-repair function of livers.

DEB-TACE has been illuminated to be at least as tolerable as cTACE in previous studies, and the adverse events are mostly low grades [16, 32]. And to some extent, DEB-TACE is even better tolerated than cTACE due to no Doxorubicin related systemic toxicity has been observed among patients treated by DEB-TACE [33]. A previous study reports that 7 (13.7%) out of 51 patients that present with complications after DEB-TACE treatment, including decompensation, hepatic vein thrombosis, pancreatitis and post embolization syndrome, the incidence of complication is relatively low [17]. In this present study, the most common adverse events during and post treatment were pain, vomiting, hypertension and fever, among which most of them were light and moderate, which was in consistent with previous studies [16, 17, 32, 33]. Those results in previous studies and ours both demonstrated a good safety by DEB-TACE treatment.

Some limitations still existed in our study: (1) the follow-up time is short, thus long-term efficacy such as overall survival was not analyzed in this study. (2) Most patients (97%) in this study had treatment history, so the efficacy and safety of CalliSpheres® DEB-TACE in treatment-naïve HCC patients could not be evaluated. (3) The sample size in our study was relatively small, which is mainly due to that the DEB-TACE treatment and the new product used in our study, the CalliSpheres® beads, are still not widely applied in clinical practice in China.

## Conclusions

In conclusion, DEB-TACE by CalliSpheres® was efficient and well tolerated in Chinese HCC patients, and BCLC stage, number of nodules and cTACE history were possibly correlated with treatment response.

## Abbreviations

AASLD: American Association for the Study of the Liver Diseases
AFP: Alpha fetoprotein
ALB: Albumin
ALP: Alkaline phosphatase
ALT: Alanine aminotransferase
ANC: Absolute neutrophil count
AST: Aspartate aminotransferase
BCLC: Barcelona Clinic Liver Cancer
BCr: Blood creatinine
BMI: Body mass index
BUN: Blood urea nitrogen
CA199: Carbohydrate antigen199
CEA: Carcino-embryonic antigen
CR: Complete response
CT: Computerized tomography
cTACE: conventional TACE
CTCAE: Common Terminology Criteria for Adverse Events
DEB-TACE: Drug eluting beads transarterial chemoembolization
ECOG: Eastern Cooperative Oncology Group
HB: Hemoglobin
HCC: Hepatocellular carcinoma
mRECIST: modified Response Evaluation Criteria in Solid Tumors
MRI: Magnetic resonance image
ORR: Overall response rate
PD: Progressive disease
PLT: Platelet
PR: Partial response
RBC: Red blood cell
SD: Stable disease
TACE: Transarterial chemoembolization
TBA: Total bile acid
TBIL: Total bilirubin
TP: Total protein
VAS: Visual analogue scale
VPCT: Volume perfusion CT
WBC: White blood cell
